# Complex Interventions and Interorganisational Relationships: Examining Core Implementation Components of Assertive Community Treatment

**DOI:** 10.5334/ijic.3547

**Published:** 2018-12-06

**Authors:** Magnus Bergmark, Ulrika Bejerholm, Bengt Svensson, Urban Markström

**Affiliations:** 1 Department of Social Work, Umeå University, Umeå, SE; 2 Department of Health Sciences, Lund University, Lund, SE

**Keywords:** integrated care, mental health services, evidence-based practice, Assertive Community Treatment, implementation, case management

## Abstract

**Introduction:**

There is increasing interest in implementing evidence-based integrated models of care in community-based mental health service systems. Assertive Community Treatment (ACT) is seen as an attractive, and at the same time challenging, model to implement in sectored service settings. This study investigates the implementation process of such an initiative.

**Methods:**

Interviews were conducted with ACT team members, the process leader, steering group members, and collaboration partners. The “Sustainable Implementation Scale” helped to identify critical implementation components, and these were further explored using the qualitative interview data. The “Tool for Measuring Assertive Community Treatment” addressed programme fidelity, and the initiative’s sustainability was assessed.

**Results:**

High-fidelity implementation of ACT in a sectored service setting is possible. Prominent components that facilitated implementation were careful preparations, team members’ characteristics, and efforts by the process leader and the steering group to improve networking. Implementation was hampered by conflicting goals among the involved authorities and a mismatch between the ACT model’s characteristics and existing organisational traditions and regulations.

**Discussion and Conclusions:**

Reducing the uncertainty caused by conflicting goals is an important step in improving the implementation of ACT. In order to facilitate implementation, the goals, regulations, and availability of resources should be aligned horizontally and vertically through the involved organisations.

## Introduction

The development of modern community-based mental health services (CMHS) has been characterized by recovery-oriented approaches, person-centred care, and the implementation of evidence-based practice (EBP) in routine treatment settings [1,2]. In this context, EBPs are usually described as well-defined interventions for which there is consistent scientific evidence showing that they produce better client outcomes compared to alternative treatment or to no treatment at all [3,4]. In many cases, EBPs are delivered as programmes. There is an increasing interest in the implementation of integrated evidence-based programmes in sectored mental health settings, but collaboration difficulties have been reported [5,6]. In order to achieve effective implementation, the planning and preparations before the start of EBP programmes have been reported important [7,8].

Jensen, Johansson, and Löfström have used the concept of interactional uncertainty to contrast different ways in which relationships either constrain or enable organisations to accomplish their tasks [9]. Interactional uncertainty can arise in both horizontal and vertical relationships. Horizontal relationships involve actors who cooperate with each other in operational work processes in order to perform an assigned task. The vertical relationships are affected by uncertainty between principals and the contractors/managers, and such relationships are related to financing, planning, monitoring, and evaluation [10]. Common reasons for vertical uncertainty are unsupportive political behaviour, or a project organisation having more than one decision maker or stakeholder. Interactional uncertainty and the strategies chosen to handle it affect the characteristics of the task-oriented activities and the possibilities of the organisation to perform its tasks [9].

In the present study, we have used critical components found in contemporary implementation research to analyse the implementation of Assertive Community Treatment (ACT) in the highly sectored CMHS system in Sweden.

ACT is known as an evidence-based intensive case management (CM) model, designed to serve people with severe mental illness. In general, case managers are supposed to maintain contact with the service users, assess their needs, and assure that these needs are met [11]. In addition, *intensive* CM usually involves high-intensity input and small caseloads (20 clients or less) [12]. The ACT model consists of a number of elements, and if these are included the intervention can be referred to as an ACT programme. ACT has its origin in the “Training in community living” treatment programme described by Test, Stein, and Weisbrod in 1980 [13,14,15]. Since it was originally published, the ACT model has been widely used and developed [16]. ACT teams include a number of selected professions, such as psychiatrists, nurses, social workers and specialists in addiction treatment and vocational rehabilitation. The teams provide support in medications, housing, finances, and everyday life problems, and some experts have highlighted 24-hour availability as an important ingredient [17].

According to several randomised controlled trials from North America, Europe, and Australia, ACT is more effective compared to treatment as usual when it comes to reducing psychiatric hospital use, homelessness, and drop-out from treatment and to improving chances for employment [18]. It has been argued that the more closely CM programmes follow the ACT principles (i.e. there is high fidelity to the model), the better the outcomes [19]. The results of different trials have been inconsistent, however, and the generalisability outside the US has been questioned. Several studies have found no evidence for ACT being more effective compared to less intense CM interventions [20,21,22]. With regard to hospital use, Burns et al. [23] found that ACT works best in settings where the participants’ initial hospital use is high.

In addition, implementation of the model has been seen as challenging [24]. Stein and Test reported that financing and coordination of the treatment programme as major implementation barriers [13]. Despite the continuous development of the ACT model, implementation difficulties have remained. A study of 13 ACT teams in two US states identified licensing and financing support, training, technical assistance, leadership, staffing, and change culture as the most prominent dimensions of effective implementation [4].

Despite the inconsistent research results and reported implementation difficulties, ACT has been reported to be a model that is attractive to implement if it is used in a proper context and is implemented with a suitable target group, and in several western countries ACT is now seen as a basic element in CMHS.

### Case Management in Sweden

Overall, the implementation of evidence-based CMHS interventions in Sweden has followed the same pattern as in other western countries. However, the development of CM has been characterised by less intensive approaches [25]. The coordinating role of CM is seen as important in Sweden’s highly sectored service system where the responsibility for providing different kinds of support is shared between several authorities. In the CMHS, the county councils’ mental health care (MHC) facilities provide the in-patient and out-patient treatment, and the municipalities are responsible for the social, housing, and daily activity support. In addition to the CMHS, many service users also receive support from the public employment service and the social insurance agencies.

In order to provide CM, several models such as Flexible ACT [26], Resource Group ACT [27], and Integrated Care [28] have been used, in most cases applied in less intensive settings. The intensive and integrated ACT model is considered attractive but so far it has been poorly disseminated. A study of one of the few Swedish national initiatives to disseminate ACT found a “drift” away from the model’s core elements [1]. Although ACT has been established as an effective treatment model [22] and the model has been given the highest priority in national guidelines of psychosocial interventions for people with severe mental illness [29], it has not gained any stable foothold in the Swedish CMHS system.

In order to more fully understand the constituents of the core implementation components and the possibilities for ACT programme sustainability in the CMHS system, we have targeted one of the first programmes with the outspoken goal of providing high-fidelity ACT in this context. The aims of this paper are thus 1) to examine to what extent high-fidelity ACT is possible to implement in a sectored context, 2) to identify components critical for a sustainable implementation and 3) to analyse implementation challenges experienced by the involved actors.

## Methods

All procedures performed in this study were in accordance with the ethical standards of the Swedish Research Ethics Laws and the Regional Ethical Guidelines and with the 1964 Helsinki Declaration and its later amendments.

### Study Context

The implementation of the ACT team was a local initiative, in a Swedish city with approximately 300,000 inhabitants. Several years before the start of the team, a “mental health service planning group” was formed. This group consisted of representatives from several stakeholders, including the social services and the MHC. The development of CM services was one of the questions being discussed, and in line with suggestions from implementation science [7] the group conducted detailed planning work that included assessments of needs, resources, and the organisation’s climate and capacity. After a collaboration agreement between the stakeholders had been assigned, the group initiated a process aiming to build up a team that would provide ACT with high programme fidelity. The planning group was transformed into a steering group, and a previously hired CM expert was employed as the process leader and the foreman of the team that was created in 2012.

The team started up its services in a local detached from other services, but after a shorter period of time it was moved to the county council’s psychosis unit. The team consisted of the process leader, a psychiatrist, an assistant nurse, three nurses, two social workers, a peer support worker and an employment specialist. The steering group applied to the financial coordination agency for financing of the process leader, the employment specialist and training courses, and the application was approved. When entering the team, the team members kept their existing employments. Accordingly, the social workers were employed and financed by the municipality, and the psychiatrist, nurses and user specialist by the county council. The process leader held pre-service and in-service training courses in the ACT model for the team members. In addition, they had an external supervisor and specialised training courses (e.g. in Motivational Interviewing and cognitive therapy) available.

In this study, a concurrent mixed study approach [30] was used. The “Sustainable Implementation Scale” (SIS) helped to identify critical components that facilitated or hampered the implementation, and the “Tool for Measuring Assertive Community Treatment” (TMACT) addressed programme fidelity. The critical implementation components were further explored using qualitative interview data. The reason for our use of this design was to better understand the research problem by converging both quantitative (broad numeric trends) and qualitative (detailed views) data. Because the present article’s focus is set on the implementation and development of the CMHS system from an organisational and team perspective, no patient outcome data are presented or analysed.

### Studying the Implementation Process

The present study was a part of a research project in which we developed the SIS. SIS is designed to identify the presence of critical implementation components and to predict organisational sustainability. The SIS implementation components are aggregated from four frameworks and reviews that have had a significant impact in the field of implementation science [7,31,32,33]. The scale and the development and testing of it, is described in detail in a previously published paper [34]. The SIS consists of 24 items sorted under the sub-scales “Components at the organisational level”, “Components at the team level”, and “Continuous strategies for support”. The following three response categories are used in the assessments of the items: *not in place* = 1 point, *partly in place* = 2 points, or *fully in place* = 3 points.

Data for assessment of the implementation process were collected one and two years after the introduction of the programme. Semi-structured interviews were performed with several categories of people involved in the programme’s implementation, including the process leader, team members, and the steering group. Some of the interviews were performed individually, and some were in groups (Table 1).

**Table 1:** Informants in the ACT implementation study.

Role	*n* after one year	*n* after two years

Process Leader	1	1
Team members	4 (2 interviews)	8 (2 interviews)
Steering group	5 (2 interviews)	5 (3 interviews)
Financial Coordination Agency	1	–
*Total*	*11*	*14*

Because the team members and the process leader had the most information to provide, these interviews were the longest (approximately 1 h 45 min each). The other interviews lasted for 30–60 minutes. The interviews were recorded and thereafter transcribed verbatim. The results from the interviews were used to conduct the SIS assessments and were used for the qualitative analysis that covered the informants’ own views of the most prominent facilitators and barriers to the implementation.

For the qualitative analysis of the implementation process, a directed content analysis model was used [35]. The initial coding categories corresponded to the implementation components in the SIS.

### Programme Fidelity Data Collection and Assessments

The ACT programme fidelity assessments followed the instructions found in the TMACT [36], and the assessments were performed at 6, 18, and 24 months after the programme was started. The data collection involved a mix of sources (chart review, daily team meeting observations, and interviews with the group leader, psychiatrist, nurses, and users). The TMACT consists of 46 items divided into the six sub-scales Operations and Structure, Core Team, Specialist Team, Core Practices, Evidence-Based Practices, and Person-Centred Planning Practices. Each item is rated on a five-point response format. According to the developers of the TMACT, mean scores above 4.0 indicate substantial adherence to the components of ACT. Two researchers (XX, XX) performed the assessments independently. Thereafter they interpreted the results in connection with the item description to facilitate the validity of the assessments. In addition, the results of the assessments were provided to the team.

### Assessment of sustainability

In order to assess the sustainability of the team, the process leader of the ACT team was contacted once again at three years after the programme’s start. Selecting a definition of sustainability can be challenging [37]. Most implementation studies do not include an operationalised definition of the term, and the existing definitions are inconsistent [38]. Because the aim of the present study relates to the possibilities of implementing the ACT model at an organisational level in a sectored health care system, we have based the assessment of sustainability on Wiltsey Stirman et al. [39], who suggest that a programme may be considered to be sustained at a given point in time if the core elements are maintained and adequate capacity for continuation of these elements is maintained after the initial implementation support has been withdrawn. The operationalised criteria we used for assessing the implementation of the ACT team as sustainable was that it should, via a formal decision of the responsible officials, be a locally funded and well-established part of the local CMHS and have good programme fidelity.

## Results

The following sections start with a presentation of the results of the assessments of the implementation components, and is followed by the programme fidelity results. Finally, the analysed qualitative interview data are presented in free text.

### Implementation Components

On the SIS-scale, the team achieved a total score of 67 out of 72 points, and the result was the same after one and two years (Table 2). The following section describes the components from each sub-scale that were most important for the implementation result.

**Table 2:** SIS result for the ACT team after one year.

Sub-scale	Score, ACT Team (*Total*)

Organisational Level	35 (*36*)
Team Level	18 (*21*)
Continuous strategies for support	14 (*15*)
Total Scale	*67* (*72*)

#### Organisational Level

The ACT team scored 35 out of 36 points. Several components that, according to previous implementation studies, are considered difficult were in place in the ACT team, including supportive management and political decisions, the organisation’s implementation climate, and traditions of collaboration. The only component that team did not score the maximum of three points was the model’s organisational fit, something that indicates a need for further adaptions of either the model that is being implemented or the organisation.

#### Team Level

The score for this sub-scale was 18 out of 21 points, meaning that most of the components were assessed as being fully in place. During the interviews, the team members’ formal qualifications were described as important factors, as was their willingness and dedication to find their roles, to work hard, and to collaborate with each other. The process leader’s qualities were highlighted by many informants because she had gained a lot of knowledge from earlier experiences of ICM and at the same time was a person dedicated to the development of the team and the ACT model.

Three SIS components in this sub-scale were assessed as “partly in place” (meaning that the functions are in place, but there is still some room for improvement): Feedback to financiers, supportive collaboration partners, and continuity among staff. The team continuously gave feedback to the steering group, but the members of the steering group did not always communicate the information to their respective managers. The collaboration with the MHC worked well. With the Social Service unit, the collaboration was more diffuse due to the complex organisation of the unit and because of the traditions of the sheltered housing intervention that overlapped the ACT services and made it difficult to decide which responsibilities the respective agency should have in each patient case. Staff continuity was disrupted because of parental leaves, sick leaves, and staff turnover during the first year. Staff turnovers are seen as risky for the implementation result because they force the team to start over and re-do critical steps in the implementation process [40].

#### Continuous strategies for support

For this sub-scale, the team’s score was 14 out of 15 points. This means that the support was satisfactory and included the availability of training courses in the model, proper supervision, and time for reflection. The only component that was not given the maximum score was administrative support.

### ACT Programme Fidelity

The ACT programme fidelity is presented in Table 3. A score of 4 points or more is considered to be good programme fidelity.

**Table 3:** ACT programme fidelity after 6, 18, and 24 months.

	6 months	18 months	24 months

Operations and Structure	3.9	4.2	4.6
Core Team	3.3	4.4	4.0
Specialist Team	2.6	4.2	4.9
Core Practices	3.6	4.0	4.0
EBP	3.6	4.1	4.4
Person-Centred Planning Practices	2.2	3.2	4.2
Total Mean Score	3.2	4.02	4.35

The sub-scale *Operations and structure* includes questions about a team’s organisation, routines, and collaborations with other agencies. Because the team continuously improved the internal and external collaboration, including the development of routines for admissions and discharges, the relatively high score at the 6-month follow-up had increased further at the second and third assessments.

*Core Team* assesses a team’s availability and the functioning of its key staff. At the 6-month follow-up, the team’s psychiatrist had limited time because of other duties, something that had been regulated at the 18-month follow-up. The employment of a secretary increased the score for the process leader because she was then able to focus more on her duties in implementing the programme instead of focusing on administrative tasks. At the 24-month follow-up, the team had all of the desired functions in place, but the TMACT score was decreased because of sick leaves and staff turnovers among the nurses.

*Specialist Team* assesses a team’s inclusion of specialists in the areas of addiction, employment, and user interests. These roles evolved during the team’s first years, and at the 24-month follow-up the functions were as prescribed by the TMACT.

In accordance to the ACT principles of *Core Practices*, the team had frequent contacts with their users at locations where the users were living their every-day lives. The support for the patients’ social network and the 24-hour support were not fully met by the team.

The sub-scale *Evidence-Based Practices* assesses the direct provision and quality of specialised services performed by the full team. Initially the team had deficiencies concerning both the magnitude and quality of services, but there were improvements over time. At the 24-month follow-up, all relevant types of treatment, except psychotherapy, were included in the team’s services. Psychotherapy was provided by external resources.

*Person-Centred Planning Practices* assesses core practices that facilitate recovery. The score was rated relatively low at the 6-month follow-up, and the team considered the treatment plans to be an item to prioritise for improvements. At the 18-month and 24-month follow-ups, the treatment plans had been partly improved by the team members.

#### Summary of the ACT Fidelity Assessment

Between the follow-ups, the team had performed continuous and successive development activities, resulting in team functions and services becoming more operationalised to reach a higher programme fidelity. The shortcomings in the 24-month follow-up were primarily related to limitations in the administrative support, crisis response capacity, and effective support in housing. Despite these shortcomings, this was considered to be a team that followed the principles of the ACT model with high fidelity.

### Implementation Challenges Experienced

The ACT team had high scores according to both the SIS and the TMACT assessments, results that indicate an effective implementation. An additional good result was that the team, at the three-year follow-up, had sustained as a regular part of the local CMHS system. However, the development of the team did not follow a straight line, and from several points of view the final implementation result was an effect of peoples’ dedication and abilities to overcome barriers more than of favourable conditions. The following section presents these barriers and the team’s strategies to overcome them, according to the results from the qualitative material.

The results of the SIS and TMACT assessments illustrated deficiencies concerning internal and external collaboration and routines, and the qualitative material provided further understanding of how these deficiencies hampered the implementation process.

Perhaps the most prominent implementation barrier concerned the social workers’ roles in the team. According to legislation there is professional secrecy between the municipality (the social workers’ employer) and the county council (the psychiatrist’s and nurses’ employer). For the team, the secrecy caused several barriers to effective implementation. One such barrier was that the social workers did not have access to the computers in the team’s office, since it was located in a county council-run building. Like in most offices, the team members employed by the county council used their computers for several purposes, such as to receive and send e-mails, to use the internet phone system, to print papers, and to write and read the patients’ health care records. The social workers could do none of that. According to regulations, it is mandatory to write patient records, and the team’s strategy to fulfil this demand without violating the secrecy was to let the social workers write their notes by hand, then hand over the papers to a secretary who wrote them down in the computer, and finally let the process leader sign the records. This strategy was considered to be complicated and time-consuming, and when the secretary had received the papers, the social workers did not have access to them anymore according to the secrecy legislation.

One of the social workers described the situation as follows:

“Sometimes I don’t get information when some of my patients stay at the hospital or have to visit the psychiatric emergency ward, which is a problem. Another problem is that I don’t have the possibility to read the patients’ case histories and plan my work in accordance to them.”

The team strived for a better solution to overcome this barrier, and they discussed this with the steering group and a legal adviser, but they found it impossible to make any better arrangement without breaking the law.

Another barrier was that the social workers were not delegated to perform any exercise of authority, meaning that they were not able to take any decisions about the participants’ social welfare benefits or housing support. Because of this, the social workers found it difficult to actualise their full potential in their work.

As shown by the result in the TMACT, the team’s possibilities to provide 24-hour crisis support was limited, a limitation that lowered the score on the SIS component “organisational fit” as well. According to working time regulations, the team members were not allowed to work at nights or on weekends as required for the highest score in the TMACT, and in addition there was no possibility to hire more staff for that task.

Psychiatrist:

“We, the team, wanted to provide services during weekends and nights, but the only strategy possible was to work overtime and get compensation for that. Neither the trade union nor our employers liked that strategy, so they tried to make a 24/7 schedule for us.”

Social Worker:

“But if we had started working according to that schedule, we would have lost a lot of other ACT elements, like the team approach…”

Psychiatrist:

“So now they have said that it is ok for us to work overtime if it concerns very important and limited efforts. So, our availability is limited and we do not have any standby duty.”

An additional barrier concerned the implementation of the “full responsibility” approach for the services that high-fidelity ACT teams are supposed to have. Many of the team’s patients were living in different types of municipality-run sheltered residential services, and received different types of additional support from them. According to the informants, these arrangements made it difficult to find out which services should be provided by the team and which should be provided by the housing units. This is in line with the assessment of the SIS component “supportive collaboration partners” as being partly in place, and according to the team members they had to put a lot of effort into negotiating with each housing unit in order to clarify areas of responsibility.

To summarise, the high scores on the implementation and fidelity scales did not come easy, and the team members considered regulations and organisational traditions as barriers that they had to fight hard to overcome. This drained them of a lot of the energy that they would have rather put into task-oriented activities with the service users. The team members described how a lot of their ability to overcome barriers was the result of hard work, dedication, and a willingness to work together as a team:

“All of us are personally engaged in this way of working, and I think this engagement and our drive and will to make this work is very important. Of course, we meet speed bumps and sometimes we drive off the road, but still we get back on track and work hard to solve the problems. And we receive a lot of support in team building activities from our process leader. I think that’s important, because it has not been easy all the way.”

The process leader’s role was something that many of the informants highlighted as an important facilitator for the successful implementation of the team. Her role was multi-faceted. She was the foreman of the team, she worked as a champion for the ACT model, and she participated in the steering group meetings where she provided feedback about the team’s development to the managers and financiers. Several informants reported that collaboration between the team and the steering group was important in order to avoid contradictory expectations among the stakeholders. Because the team members had different managers in their host organisations, the process leader also had to keep in contact to them in order to discuss questions related to human resources. During the interviews, the staff and the steering group members expressed the process leader’s efforts at different organisational levels as a critical ingredient for the development of the team.

## Discussion

This study shows that, despite the model’s complexity and a highly sectored CMHS system, a sustainable implementation of high-fidelity ACT services is possible. This is an interesting result compared to previous studied initiatives, where the ACT core components drifted [1].

Overall, the ACT elements applying to the team members’ interventions with the users (like the team and outreach approach) were seen as possible to adapt to the specified context. The ACT elements considered as difficult to implement in most cases applied to the model’s organisational fit or collaboration structure (for example the 24-hour crisis support and the administrative support to the social workers). These elements relate to the components found in the SIS sub-scale ‘Components at the organisational level’, and the importance of those is in line with the results of previously conducted implementation studies of integrated models in the selected sectored CMHS system. For example, a study of 14 “Individual Placement and Support” programmes for vocational rehabilitation of people with mental illness pointed out the preparation of the programmes as critical for the programmes’ sustainability [8], and according to a study of seven “flexible ACT” programmes, good SIS results could be explained by an active national initiative and the team’s access to implementation support [41].

The ACT team in the present study was not nationally initiated, but the carefully conducted planning and anchoring of the team seems to have been important for the successful implementation result. This is in accordance with the framework produced by Meyers et al. [7], which suggests that most of the critical steps in the implementation process have to be taken before the actual implementation begins. One such critical step was the deliberate composition of the team, which assured that the team members had competences relevant for the task. Maybe even more critical for the implementation result was the team members’ willingness and dedication to carry out the change process that is involved in building a new team with new ways of working and new ways of networking [33].

According to Provan and Milward [42], interorganisational networks can lead to improved system-level outcomes, but only if the resources are adequate, the system is stable, the external control is direct and nonfragmented, and the network integration is centralised. The team in the present study was able to acquire adequate resources, but they were negatively affected by deficiencies related to the three other prerequisites. Several system-level barriers clearly complicated the team’s interorganisational networking. For example, the regulations concerning working hours, the secrecy regulations, and the fact that the team members did not have the same preconditions because they were not employed by the same authority were all seen as barriers that neither the staff, the process leader, nor the steering group had any possibilities to fully influence in their desired directions. In addition, the members in the steering group had to consider the needs of each of their host organisations, which sometimes were conflicting with the team’s needs. These kinds of conflicting goals forced the team members and steering group to put a lot of time and energy into networking activities and negotiations instead of task-oriented activities such as the clinical work with the users [9].

An additional barrier was that the team’s collaboration partners considered the ACT model’s organisational pattern to be deviant [12], and that it changed the circumstances for them [43]. For example, the services provided by the already existing sheltered housings were fully in line with the intentions of the mental health reform and local ambitions, but not with the ACT team’s full-range service ambitions. Thus, the implementation of ACT involved a tension between existing norms and high model fidelity. Such tensions might stress the interorganisational network and increase the interactional uncertainty [9].

Leadership has been described as a critical factor for high fidelity implementation of ACT, especially concerning the organisational level [4]. In the team studied, the process leader and steering group’s discussions contributing to information sharing and to creating shared visions were critical. Because implementation is a social process and implementation results are dependent on the creation of social networks and the quality of formal and informal communications [32], the importance of these actions should not be underestimated. In order to facilitate the interorganisational networking, the process leader worked as a link between the SIS levels of “team” and “organisation”, which was important for both of the levels because it supported feedback and collaboration between the levels.

**Figure 1:**
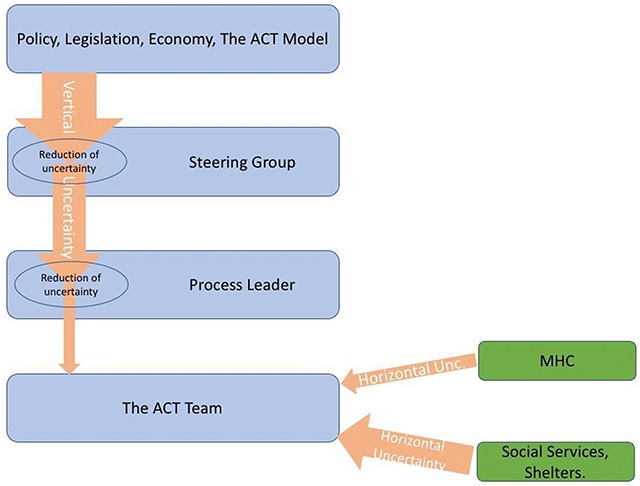
Examples of flows of interactional uncertainty, loosely based on Jensen et al. [9].

In the tentative model (Figure 1), we have illustrated the interactional uncertainty affecting the team (arrows).

The reduction of the vertical arrows’ width illustrates that the steering group and the process leader reduced some of the vertical uncertainty and thereby protected the team from it. Several strategies were used by the steering group members in order to reduce the vertical uncertainty. They conducted a juridical investigation and they discussed rules and legislation with local politicians and in addition they held meetings with the process leader where they discussed important issues and exchanged feedback. The process leader in her turn provided supervision and foremanship to the team. The horizontal uncertainty was lower in the relationship with the MHC compared to the relationship with the Social Services and the shelters, where the unclear collaboration hampered the implementation.

Assessments and analyses of implementation components and programme fidelity are useful perspectives for increasing the understanding of the implementation of integrated EBP interventions in a sectored CMHS field. At the same time, this type of implementation includes several inherent complexities that are difficult to grasp. In the development of a team with several stakeholders, a circumstance that is considered beneficial for one part might be regarded as detrimental for another part. Therefore, monitoring the effect of the implementation process on the involved organisations and on the level of uncertainty is an important challenge for programme stakeholders.

Another implication for stakeholders is that if high programme fidelity is the goal, the negotiations of objectives and forms for collaboration and responsibility areas should be held at the highest organisational level possible in order to protect the teams from conflicting goals and interactional uncertainty and thereby allow them to put most of their energy into task-oriented activities. From society’s perspective, and according to national goals, the type of integrated and intensive CM that ACT represents seems favourable, but it is likely that all organisations involved would have to gain benefits from such initiatives in order to decrease vertical uncertainty and facilitate implementation and organisational sustainability.

The need to effectively coordinate services across sectors is well documented [44], but integrating the social services with the MHC has been reported to be difficult [25]. Preparations and strategic networking, support from the steering group and the process leader, and a staff with endurance were all critical for the successful implementation seen in this study. However, if organisational sustainability is highly dependent on the staff’s and process leader’s dedication, as it is in the case studied here, it will be risky to release resources for these types of activities without simultaneously making investments in the form of incentives for networking and the provision of active implementation support at the national level.

### Limitations and Methodological Considerations

In this study, the SIS and TMACT assessments were conducted by the same researchers, an arrangement that includes the risk of cognitive bias because a positive impression of one test might influence the researchers’ overall opinion of the research object [45]. However, we believe that our strategy to triangulate data and use multiple informants and data sources has reduced such bias.

It can be seen as surprising that the SIS score was the same at one and two years after the start of the programme, but this result illustrates both benefits and shortcomings with the scale. The three-point response format used in the SIS makes it a useful tool for getting an overall picture of a team’s implementation, and it has the capacity to predict sustainability [34]. At the same time, however, the scoring procedure is quite rough such that the scale does not catch minor organisational changes. In addition, many of the components that are assessed are related to the start of a team (e.g. recruitment of staff and experiences of similar models). Probably the greatest strengths with the SIS is the ability to use it as a check-list at the start of a new team and as an assessment tool up to one year after the start of the programme.

A limitation with the single case study approach is that we did not have the possibility to compare this ACT initiative to any analogous teams. Instead, we have analysed the team on the basis of contemporary international research about barriers and facilitators to implementation generally and to ACT specifically. This approach made it possible to study circumstances specific for both the model and the context. With the insights gained from combining these approaches and by adding the perspectives of networking and interorganisational relationships, we believe that we have contributed to useful insights concerning the importance of alignment of all levels involved in implementation of evidence-based, integrated models. As the saying goes, a chain is only as strong as its weakest link, but because implementation is far more complex than a traditional chain, further research is needed in order to learn how to identify and strengthen the weak links. There is still a need for studies with larger numbers of cases included, and there is also a need for further research on how the political, system, and network levels can facilitate development, collaboration between involved authorities, and implementation of integrated evidence-based interventions in the field of CMHS.
